# Discovery of Tricyclic Aromatic Polyketides Reveals Hidden Chain-Length Flexibility in Type II Polyketide Synthases

**DOI:** 10.3390/ijms26167801

**Published:** 2025-08-13

**Authors:** Yao Liu, Lijun Wang, Haiyan Wang, Yuchen Zhu, Jianing Sun, Boyang Ma, Lin Liu, Xunrui Bao, Jinwei Ren, Keqiang Fan, Liyan Wang, Xiao Li, Guohui Pan

**Affiliations:** 1 State Key Laboratory of Microbial Diversity and Innovative Utilization, Institute of Microbiology, Chinese Academy of Sciences, Beijing 100101, China; 2 University of Chinese Academy of Sciences, Beijing 101408, China; 3 College of Chemistry and Life Science, Beijing University of Technology, Beijing 100124, China; 4 School of Chemical & Environmental Engineering, China University of Mining and Technology (Beijing), Beijing 100083, China

**Keywords:** type II polyketide synthases, chain length, nonaketide, aromatic polyketides, biosynthesis

## Abstract

Type II polyketide synthases (PKSs) collectively generate polyketide intermediates of varying chain lengths, which undergo cyclization and further tailoring to produce structurally diverse aromatic polyketides. The length of the polyketide chain is a critical factor shaping the core scaffold of the final product. However, individual type II PKSs typically produce intermediates with a fixed chain length, thereby limiting the structural diversity accessible from a single biosynthetic system. In this study, we report the discovery of two pairs of novel tricyclic aromatic polyketides, varsomycin C/C′ and oxtamycin A/A′, along with two known analogues. These compounds are derived from the *var* and *oxt* gene clusters in *Streptomyces varsoviensis*/*varR1*, which primarily produce decaketide-derived tetracycline natural products, varsomycin A-B and oxytetracycline. Bioinformatic analysis combined with metabolite profiling of gene-disrupted mutants indicated that varsomycin C and C′ are co-produced by enzymes encoded in the *var* cluster, with contributions from *oxtJ* and *oxtF* in the *oxt* cluster, resulting in nonaketide-derived tricyclic scaffolds. Oxtamycin A and A′, along with the two analogues, are predicted to be biosynthesized by the *oxt* cluster. These results suggest that the minimal PKSs from both clusters possess intrinsic flexibility in controlling polyketide chain length, enabling the production of both decaketide and nonaketide intermediates, which represents a rare example of dual chain-length programming in type II PKSs. This flexibility reveals new natural sources of nonaketide biosynthetic enzymes and enriches the chemical diversity of tricyclic aromatic polyketides. Our findings deepen the understanding of type II PKS chain-length regulation and provide a foundation for future engineering of PKSs to produce customized bioactive aromatic polyketides.

## 1. Introduction

Bacterial aromatic polyketides represent a large and structurally diverse family of natural products, many of which possess remarkable pharmacological properties, including antibiotic, anticancer, and immunosuppressive activities [1,2]. Classical examples include tetracyclines (e.g., tetracycline, oxytetracycline, and chlortetracycline) [3,4], which have led to the development of broad-spectrum antibiotics, and anthracyclines [5] (e.g., daunorubicin [6]), which have served as scaffolds for clinically important antitumor drugs such as doxorubicin and epirubicin [7,8]. These polycyclic aromatic scaffolds are primarily synthesized by type II polyketide synthases (PKSs), which construct polyketide backbones through iterative decarboxylative condensations of malonyl-CoA extender units [9,10]. The minimal PKS, comprising a ketosynthase (KS), chain length factor (CLF), and acyl carrier protein (ACP), produces ACP-tethered linear poly-*β*-ketone intermediates of defined chain lengths. These intermediates undergo various cyclization modes, mediated by cyclases/aromatases and auxiliary enzymes, to form diverse aromatic frameworks [11,12,13]. Subsequent modifications by a wide array of tailoring enzymes, such as oxidoreductases, glycosyltransferases, and methyltransferases, diversify the core scaffolds into structurally complex aromatic polyketides [14,15,16,17].

**Figure 1:**
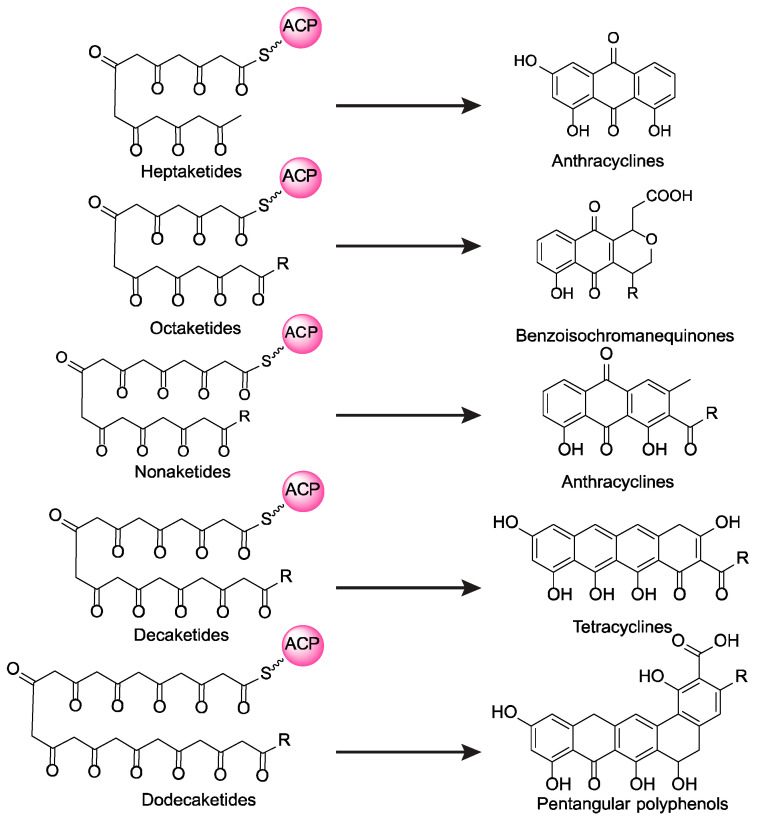
Structural diversity of aromatic polyketides driven by different chain-length intermediates produced by type II polyketide synthases.

Typically, each type II PKS generates polyketide intermediates with a specific, fixed chain length, with decaketides (C20) being the most common [18]. These decaketides account for approximately 60% of all known type II PKS products and serve as key precursors for major natural product families, including tetracyclines, anthracyclines, and angucyclines [19]. In contrast, a smaller number of type II PKSs produce intermediates with alternative chain lengths. For example, heptaketides (C14) lead to tricyclic anthracyclines, octaketides (C16) give rise to benzoisochromanequinones, nonaketides (C18) result in tricyclic anthracyclines with distinct structural features, and dodecaketides (C24) are precursors to pentangular polyphenols (Figure 1) [13,20]. This chain-length specificity is generally attributed to CLF, a core component of the minimal PKS, which controls intermediate elongation through steric constraints within the substrate-binding tunnel [10,21,22]. While individual minimal type II PKSs typically produce polyketide intermediates with a single, defined chain length, rare cases have been reported in which a single PKS generates intermediates of variable lengths [23,24,25]. For instance, in the biosynthesis of oxytetracycline, whose type II PKS normally synthesizes a decaketide intermediate, the disruption of *oxyS* (encoding an anhydrotetracycline oxygenase) resulted in the production of a novel tricyclic aromatic polyketide (compound C1-1) [25]. This observation implies that the type II PKS may have also generated a nonaketide intermediate under an altered enzymatic context. These findings suggest that some type II PKSs may possess an intrinsic but underappreciated potential for chain-length flexibility.

In the present study, we investigated the metabolic potential of *Streptomyces varsoviensis* NRRL ISP-5346/*varR1*, which harbours two type II PKS gene clusters: *var* and *oxt* [26]. Our previous work established that these clusters primarily synthesize decaketide-derived products, including varsomycins and oxytetracycline [26]. Interestingly, detailed metabolomic and structural analysis revealed six aromatic polyketides, including two pairs of novel tricyclic aromatic polyketide enantiomers (compounds **3**–**6**) and two known analogues (compound **7**, a tricyclic scaffold; and compound **8**, a tetracycline analogue). Bioinformatic analysis, along with metabolite profiling of gene-disrupted mutants, suggests that compounds **3** and **4** are biosynthesized by enzymes encoded in the *var* cluster, with contributions from *oxtJ* and *oxtF* of the *oxt* cluster, leading to the formation of nonaketide-derived tricyclic aromatic scaffolds. Compounds **5**–**8** are likely biosynthetic products of the *oxt* gene cluster. This discovery provides evidence that the minimal PKSs encoded by *var* and *oxt* are capable of generating both decaketide and nonaketide intermediates. Our findings thus uncover two previously unrecognized type II PKSs with dual chain-length programming capabilities, expanding the known scope of natural sources for nonaketide-derived polyketides. These results not only deepen our understanding of type II PKS programming and chain-length control but also provide potential support for the future rational engineering of PKSs to produce custom-length intermediates for novel bioactive aromatic polyketides.

## 2. Results

**Figure 2:**
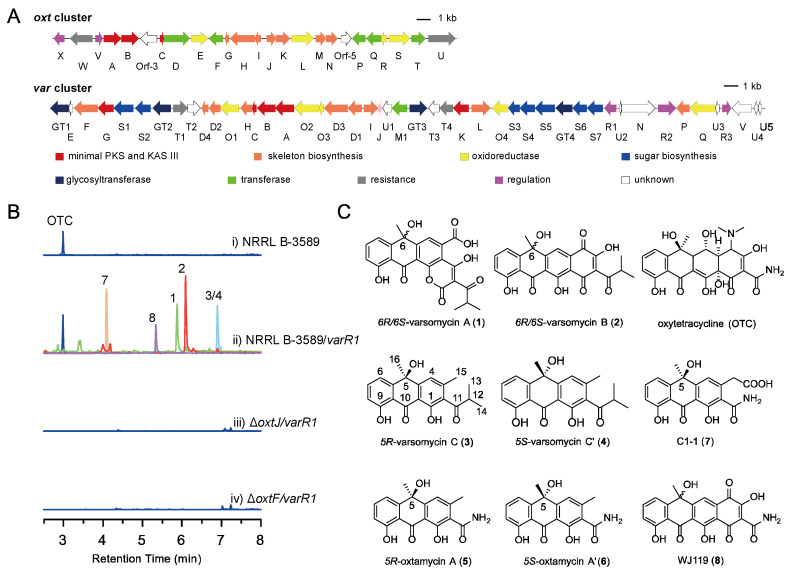
Overview of the *var* and *oxt* gene clusters and their metabolic products. (**A**) Schematic representation of the *var* and *oxt* gene clusters. (**B**) LC–MS analysis of metabolites from wild-type NRRL B-3589 and mutant strains. (**C**) Chemical structures of compounds **1**–**8**.

In our previous work [26], we developed a targeted strategy for identifying tetracycline (TC) biosynthetic gene clusters (BGCs). Among the strains analyzed, *Streptomyces varsoviensis* NRRL ISP-5346 attracted particular interest due to the presence of two TC BGCs, *oxt* and *var*. The *oxt* cluster is highly similar to the well-characterized *oxy* cluster responsible for oxytetracycline (OTC) biosynthesis (Figure 2A). The *var* cluster was successfully activated by overexpressing an SARP family regulatory gene, leading to the appearance of new peaks in the HPLC profile and the isolation of varsomycin A (**1**) and varsomycin B (**2**). Notably, compounds **1** and **2** each consist of a pair of 6*R* and 6*S* enantiomers, resulting from spontaneous oxidation at the C6 position. Gene deletion analysis revealed that varsomycin biosynthesis utilizes an unusual isobutyryl starter unit and recruits the C9-ketoreductase OxtJ and C6-methyltransferase OxtF from the *oxt* cluster.

**Figure 3:**
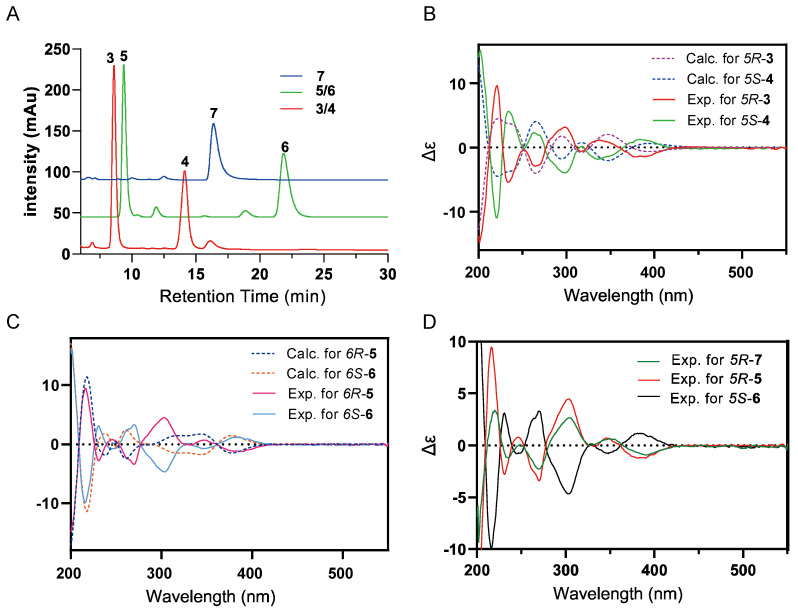
Chiral analysis and ECD spectral comparison of compounds **3**–**7**. (**A**) Chiral separation of compounds **3**/**4**, **5**/**6**, and **7**. (**B**) Experimental and B3LYP/6-31+G(d,p) ECD spectra of compounds **3** and **4**. (**C**) Experimental and B3LYP/6-31+G(d,p) ECD spectra of compounds **5** and **6**. (**D**) Experimental ECD spectra of compounds **5**, **6**, and **7**.

Previously identified varsomycins and OTC are both derived from decaketide intermediates [26]. To investigate whether the two PKSs encoded by *S. varsoviensis* NRRL ISP-5346 possess the capacity to generate polyketides of alternative chain lengths, we performed a detailed analysis of the metabolic profile of the strain *S. varsoviensis* NRRL ISP-5346/*varR1*. This analysis revealed several aromatic polyketides distinct from varsomycins A and B (Figure 2B, i and ii). Compounds **3**–**8** were subsequently isolated using a combination of chromatographic techniques. Chiral HPLC analysis indicated that compounds **3**/**4** and **5**/**6**, like varsomycin A (**1**) and B (**2**), are mixtures of enantiomers. Their optically pure forms were successfully separated by chiral column chromatography (Figure 3A). Finally, the structures of all six compounds were elucidated by HRMS, NMR, and ECD spectroscopy.

Compounds **3**/**4** were isolated as co-eluting yellow powders. HR-ESI-MS analysis afforded molecular formula as C_20_H_20_O_5_ (*m*/*z* [M − H]^−^ calcd for C_20_H_19_O_5_, 339.1233; found 339.1221) (Figure S6), implying eleven degrees of unsaturation. The ^1^H NMR spectrum displayed two methyl singlets at δH 1.58 and 2.31 and two overlapping methyl doublets at *δ*_H_ 1.17, *J* = 7.0 Hz. Aromatic protons resonated at *δ*_H_ 7.63 (dd, *J* = 7.7, 8.3 Hz), 7.42 (br. d, *J* = 7.7 Hz), and 6.91 (br. d, *J* = 8.3 Hz)], constituting an ABC spin system. This pattern, together with an isolated aromatic singlet at *δ*_H_ 7.34, indicates a substituted aromatic framework. The ^13^C NMR spectrum resolved 20 carbon signals: twelve aromatic carbons (*δ*_C_ 112.6–163.8), two non-equivalent carbonyls (*δ*_C_ 212.3 and 193.4), and six aliphatic carbons, one of which was an oxygenated tertiary carbon (*δ*_C_ 71.2) (Table 1). HMBC correlations between H_3_-13/-14 (*δ*_H_ 1.17) and C-11/C-12 (*δ*_C_ 212.3 and 42.8) locate an isobutyryl group substituent. Additional key connectivities—H-4 to C-2, C-3, C-4a, C-10a and C-10; H_3_-16 to C-4a, C-5 and C-5a; H-6 to C-8 and C-9a; and H-7 to C-5a and C-9—define a tricyclic aromatic core similar to that of varsomycin A (**1**) (Figures S1–S5). These data established the planar structure of compounds **3** and **4.** Subsequent chiral-phase separation, together with a comparison between experimental and TDDFT-calculated ECD spectra disclosed that compounds **3** and **4** are a pair of enantiomers (Figure 3A,B). Finally, compound **3**, designated varsomycin C, was assigned as the 5*R* configuration, while **4**, varsomycin C′, corresponded to the 5*S* enantiomer (Figure 2C and Figure 3A). The ratio of **3** (5*R*) and **4** (5*S*) is approximately 1:1 (Figure 3A).

Compounds **5**/**6** were obtained as yellow powders. HR-ESI-MS analysis established their molecular formula as C_17_H_15_NO_5_ for both (Figure S10). The overlay of their ^1^H, ^13^C NMR, and HMBC spectra with those of **3** and **4** revealed an identical tricyclic scaffold, with the only difference being the substitution of the isobutyryl side chain in **3**/**4** with an amide group in **5**/**6** (Figures S1 and S7–S9), evidenced by the mass interpretation and the diagnostic ^13^C shift at *δ*_C_ 167.4 (NH_2_ –*CO*) (Table 1). Chiral HPLC afforded baseline-separated peaks whose ECD traces confirmed **5** and **6** as an enantiomeric pair (Figure 3A). TDDFT calculations reproduce experimental Cotton effects (Figure 3C), allowing unambiguous assignment of the 5*R* configuration to **5** (oxtamycin A) and 5*S* configuration to **6** (oxtamycin A′) (Figure 2C and Figure 3A,C). Compounds **5** (5*R*) and **6** (5*S*) are present in an approximate ratio of 4:3 (Figure 3A).

Compound **7** exhibited high structural similarity to **5** and **6**. Detailed spectral interpretation revealed that the key difference was the presence of an additional carboxyl group at the C3 position. The NMR data were consistent with those previously reported for C1-1 [25] (Figures S11–S14). The absolute configuration of **7** was determined to be 5*R* by comparing its ECD spectrum to that of **5** and **6** (Figure 2C and Figure 3D). Compound **8** possesses the same tetracyclic framework as **2**, differing only by an amide substituent at C-2. Comparison of their ^1^H and ^13^C NMR data indicated that the structure of **8** is identical to that of the known compound WJ119 [27] (Figure 2C, Figures S15, S16 and S18). The absence of a Cotton effect in the ECD spectrum of compound **8** suggests that it exists as a racemic mixture of enantiomers (Figure S17).

In our previous work [26], the *var* and *oxt* clusters were shown to collaborate in the biosynthesis of varsomycin A and B. To investigate the biosynthetic origin of **3**–**8**, we compared the LC/MS profiles of metabolites from *S. varsoviensis* NRRL ISP-5346/*varR1* and relevant gene disruption mutants. It was previously demonstrated that varsomycin A and B biosynthesis involves the recruitment of C9-ketoreductase OxtJ and C6-methyltransferase OxtF from the *oxt* cluster.

Disruption of either *oxtJ* or *oxtF* abolished the production of compounds **1**–**8** (Figure 2B, iii and iv), indicating that these enzymes are involved in the biosynthesis of compounds **3**–**8** as well. Based on this, we propose that compounds **3** and **4** are produced through the cooperative biosynthesis of both the *var* and *oxt* clusters, involving *oxtJ* and *oxtF* from the *oxt* cluster. Considering that compounds **5**–**8** share an identical amide side chain with oxytetracycline (OTC), it is likely that they are biosynthetic products derived primarily from the *oxt* gene cluster.

Compounds **3**–**8** were evaluated for antibacterial and antitumor activities. However, they did not exhibit significant antibacterial effects against tested strains including methicillin-resistant *Staphylococcus aureus* (MRSA), *Escherichia coli*, and *Acinetobacter baumannii*. Additionally, no notable antitumor activity was observed in multiple cell lines, such as HuH-7, HeLa, and HEK293T.

## 3. Discussion

**Figure 4:**
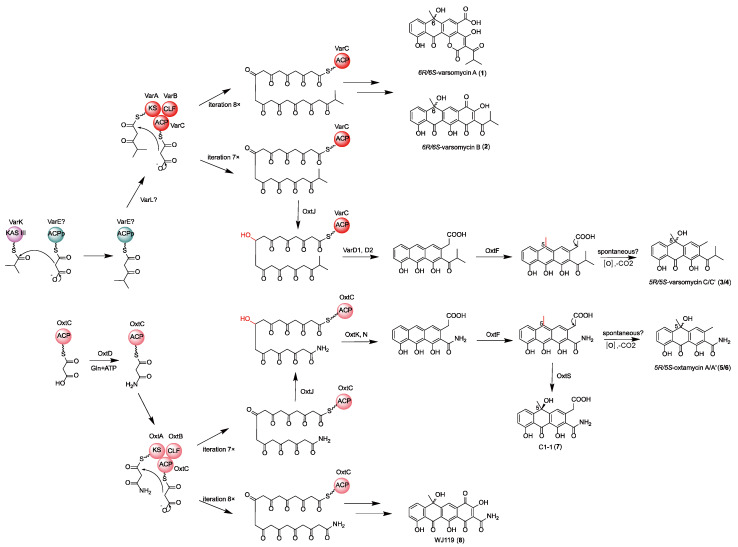
Proposed biosynthetic pathway for compounds **3**–**8**.

Based on experimental data and bioinformatic analysis, we propose the biosynthetic pathway of compounds **3**–**8** (Figure 4). The biosynthesis of compounds **3** and **4** is collaboratively carried out by the *var* and *oxt* gene clusters. The KAS III enzyme VarK catalyzes the formation of an unusual isobutyryl starter unit, which is then transferred to the minimal PKS encoded by the *var* cluster to initiate the biosynthesis of compounds **3** and **4**. This is followed by seven iterative decarboxylative Claisen condensations using malonyl-CoA as the extender unit, resulting in the formation of a nonaketide intermediate. After C9-ketoreduction catalyzed by OxtJ from the *oxt* cluster, two cyclases, VarD1 and VarD2, catalyze the formation of the tricyclic skeleton. Subsequently, OxtF catalyzes C5-methylation. Finally, compounds **3** and **4** (in approximately a 1:1 ratio) are formed through spontaneous decarboxylation and C5-hydroxylation reactions.

For the biosynthesis of compounds **5**–**8**, the amidotransferase OxtD catalyzes the formation of an amide-containing starter unit. The minimal PKS encoded by the *oxt* cluster then catalyzes seven iterative decarboxylative Claisen condensation reactions to produce a nonaketide intermediate. Similarly, under the action of OxtJ and two cyclases, OxtK and OxtN, the tricyclic aromatic skeleton is formed. Following this, C5-methylation is catalyzed by OxtF to generate an intermediate. Spontaneous decarboxylation and predominantly non-enzymatic C5-hydroxylation yield compounds **5** and **6** in an approximate 4:3 ratio, with the modest excess of the C5 *R*-configured compound **5** likely arising from OxtS-catalyzed hydroxylation of a small fraction of substrates. Additionally, this intermediate can undergo stereospecific hydroxylation at C-5 to produce a C5 *R*-configured hydroxyl group catalyzed by OxtS, resulting in compound **7**. In contrast, the biosynthesis of compound **8** requires eight iterative condensation reactions catalyzed by the minimal PKS to form a decaketide intermediate. Subsequent modifications, including C9-ketoreduction, cyclization, methylation, and hydroxylation, ultimately yield the typical tetracycline scaffold (Figure 4).

**Figure 5:**
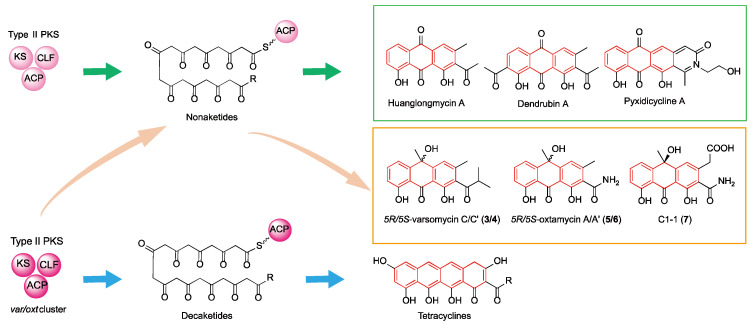
Chain-length flexibility of type II polyketide synthases encoded by the *var* and *oxt* gene clusters enables the biosynthesis of structurally diverse aromatic polyketides.

Our findings indicate that the minimal PKSs from the *var* and *oxt* clusters exhibit intrinsic flexibility in chain-length control. Besides primarily synthesizing decaketide products, they also have the capability to produce nonaketides. It is very rare for a single type II PKS to generate two different chain-length products simultaneously. Previously, a similar case was reported in the biosynthesis of oxytetracycline (OTC), where the compound C1-1 was identified in an *oxyS* deletion mutant [25]. Studies suggested that OxyS may influence the polyketide chain length through protein–protein interactions with the minimal PKS rather than through its catalytic activity, although the exact mechanism remains unclear [25]. Currently, the number of type II PKSs capable of synthesizing nonaketides is limited, accounting for only about 5% of all known type II PKSs [18]. The products derived from these nonaketide intermediates are mainly tricyclic aromatic polyketides (Figure 5). Examples include huanglongmycins [28], which exhibit significant cytotoxicity; dendrubins [29], known for notable antibacterial activity; pyxidicyclines [30], which display strong topoisomerase inhibition after post-cyclization modifications; and dimerized tricyclic compounds such as setomimycin [31] and julichromes [32], which possess antibacterial and antitumor properties. Our study not only expands the known sources of nonaketide biosynthetic enzymes but also enriches the chemical diversity of tricyclic aromatic polyketide natural products, underscoring how evolutionary flexibility in chain-length control contributes to natural product diversification.

## 4. Materials and Methods

### 4.1. General Experimental Procedures

The ECD data were collected using a Chirascan spectrometer (Applied Photophysics, Ltd., Leatherhead, Surrey, UK). High-resolution electrospray ionization mass spectrometry (HR-ESI-MS) data were recorded on a Thermo Scientific LTQ Orbitrap XL instrument (Thermo Fisher Scientific Inc., Waltham, MA, USA), and 1D and 2D NMR spectra were recorded using a Bruker Avance III spectrometer, Bruker BioSpin, Zug, Switzerland (500 and 125 MHz for ^1^H and ^13^C NMR, respectively) as the internal standard at room temperature. The HPLC analysis was performed with a linear gradient elution system from 5% to 95% acetonitrile in water containing 0.1% formic acid (FA) for 23 min at a flow rate of 1.0 mL min^−1^. The chiral HPLC analysis and semipreparation of enantiomers was performed on an Agilent 1260 system equipped with a ChiralPAK AD-H column (5 μm, 4.6 mm × 250 mm, Daicel Corporation/CPI Company, Tokyo, Japan) with VWD detector (Agilent Technologies Inc., Santa Clara, CA, USA).

### 4.2. Strains, Growth, and Fermentation Conditions

All bacterial strains in this study were derived from our previous work, and the strains were cultured on mannitol soya flour (MS) agar plates for sporulation. Fermentation of the strains was carried out in fermentation medium SSM (sucrose 8%, soybean powder 2%, skimmed milk powder 0.1%, CaCO_3_ 0.3%, K_2_HPO_4_ 0.1%, and FeSO_4_·7H_2_O 0.01%, pH 7.0) at 28 °C and 200 rpm for 7 days. To stabilize and adsorb the secondary metabolites, Diaion HP-20 resins (4%, *w*/*v*) were added to the fermentation cultures 12 h after inoculation.

### 4.3. Computational ECD Calculation

Conformational searches were performed using the Merck Molecular Force Field (MMFF) implemented in MacroModel. All DFT and TDDFT calculations were performed using the Gaussian 16 software suite. Conformers within an energy window of 12 kcal mol^−1^ were generated and optimized at the B3LYP-D3(BJ)/6-31+G(d,p) level of theory, with diffuse functions included where appropriate. Frequency analyses confirmed that all optimized conformers correspond to true minima (no imaginary frequencies) and provided the relative thermal free energies (ΔG) at 298.15 K. The selected conformers were then re-evaluated in methanol at the same level of theory, with solvent effects modelled using the self-consistent reaction field (SCRF) approach under the IEFPCM scheme [26,33].

### 4.4. Isolation and Purification of Compounds

After the fermentation was finished, the resins were collected and then extracted with methanol. Subsequently, the methanol was removed in vacuo to obtain the crude extract using a reverse-phase column packed with the C18 bulk resin (50 µm, 100 Å, QuikSep, Beijing, China) to separate the crude extract, employing a linear gradient programme from 10% to 90% (*v*/*v*, both containing 0.1% formic acid) acetonitrile in water. The fractions containing the target compounds were concentrated and further fractionated by Sephadex^TM^ LH-20 (GE Healthcare Bio-Sciences AB, Uppsala, Sweden) eluted with MeOH. Finally, the pure compounds were obtained using preparative Agilent HPLC system equipped with Shim-pack Prep-ODS(H) KIT (5 µm, 20 × 250 mm), Shimadzu, Kyoto, Japan, eluted by using acetonitrile in water with 0.1% formic acid at a flow rate of 12 mL/min unless otherwise noted.

### 4.5. Antibacterial Activity and Cytotoxicity Assay

The antibacterial activity and cytotoxicity assay procedures in this work followed established methodologies [26].

## 5. Conclusions

In conclusion, we identified two pairs of novel tricyclic aromatic polyketides, varsomycin C/C′ and oxtamycin A/A′, along with two known analogues, derived from the *var* and *oxt* gene clusters. Compounds **3** and **4** are produced via coordinated biosynthesis involving both clusters, while compounds **5**–**8** are likely synthesized by the *oxt* cluster alone. These findings reveal two rare type II PKSs capable of generating both decaketide and nonaketide intermediates, further uncovering the hidden flexibility in polyketide chain-length programming. This expands the known scope of natural sources for nonaketide-derived polyketides and enriches the structural diversity of tricyclic aromatic polyketide natural products. This study deepens our understanding of type II PKS programming and chain-length regulation and offer a basis for engineering PKSs to produce customized intermediates for new bioactive aromatic polyketides.

## Figures and Tables

**Table 1:** ^1^H NMR (500 MHz) and ^13^C NMR (125 MHz) spectroscopic data of **3**/**4** in MeOD and **5**/**6** in DMSO-*d*_6_ (δ in ppm and *J* in Hz).

	Compound 3/4		Compound 5/6	
Position	δ_H_ (*J* in Hz)	δ_C_	δ_H_ (*J* in Hz)	δ_C_
1		160.6		167.4
2		130.0		126.6
3		146.8		144.8
4	CH, 7.34, s	120.3	CH, 7.28, s	118.5
4a		153.6		151.3
5		71.2		69.4
5a		152.8		152.0
6	CH, 7.42, dd (7.7, 1.0)	118.0	CH, 7.42, dd (7.7, 0.8)	117.2
7	CH, 7.63, dd (7.7, 8.3)	138.5	CH, 7.68, dd (7.7, 8.3)	137.4
8	CH, 6.91, dd (8.3, 1.0)	117.5	CH, 6.94, dd (8.3, 0.8)	116.0
9		163.8		161.5
9a		114.5		113.0
10		193.4		191.5
10a		121.6		110.8
11		212.3		167.4
12	CH, 3.31, m	42.8	CH_3_, 2.36, s	20.0
13	CH_3_, 1.17, d (7.0)	18.1	CH_3_, 1.50, s	39.5
14	CH_3_, 1.17, d (7.0)	18.3		
15	CH_3_, 2.31, s	20.7		
16	CH_3_, 1.58, s	38.8		
1-OH			7.80, s	
5-OH			6.24, s	
9-OH			7.55, s	

## Data Availability

The data presented in this study are available in this article.

## Supplementary Materials

The following supporting information can be downloaded at https://www.mdpi.com/article/10.3390/ijms26167801/s1.
