# Supplementary material for: Discovery of Tricyclic Aromatic Polyketides Reveals Hidden Chain-Length Flexibility in Type II Polyketide Synthases
Source: Int J Mol Sci. 2025 Aug 13;26(16):7801. doi: 10.3390/ijms26167801 (PMC12386333; doi:10.3390/ijms26167801)

Figure S1. key HMBC correlations of compounds 3/4 and 5/6.

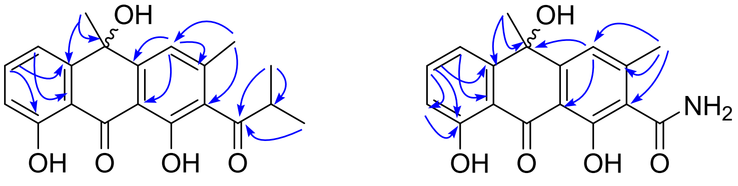

5R/5S-varsomycin C/C' (3/4) *5R/5S*-oxtamycin A/A' (5/6)

Figure S2. ^{1}H NMR spectra of **3/4** in DMSO-d6.

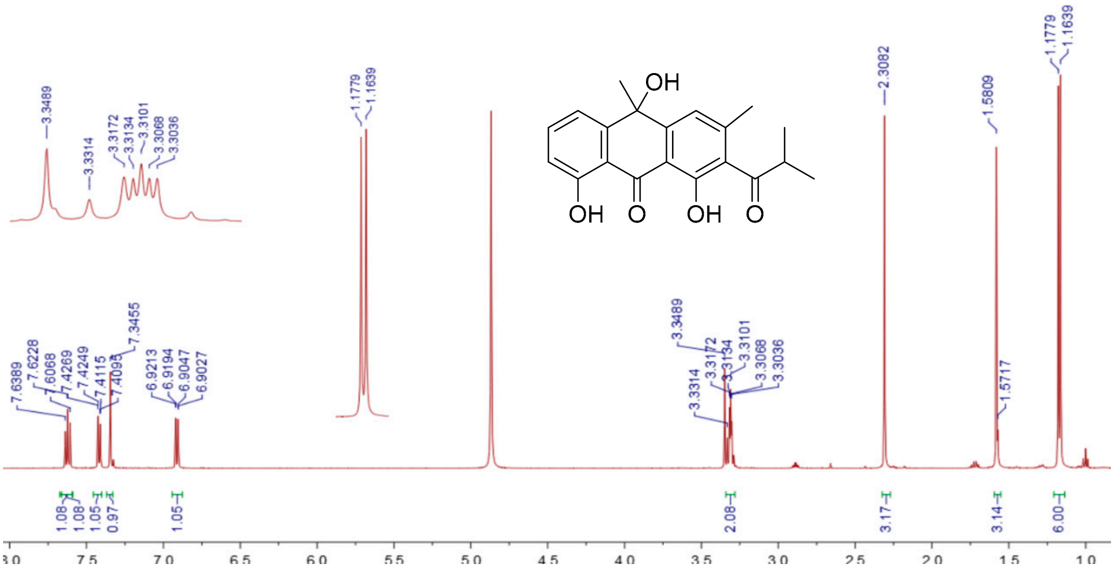

Figure S3. ^{13}C NMR spectra of **3/4** in DMSO-*d6*.

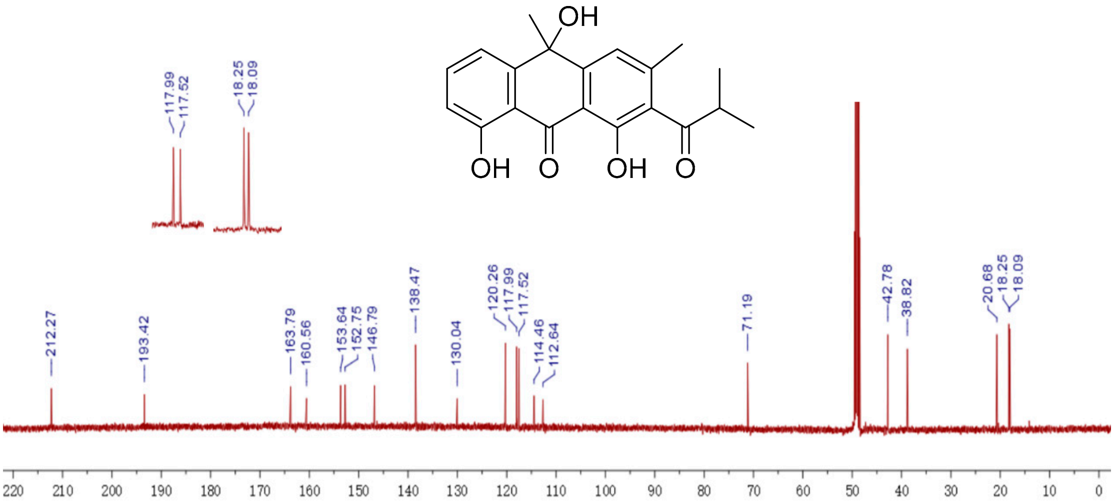

Figure S4. HSQC spectra of **3**/**4** in DMSO-*d6*.

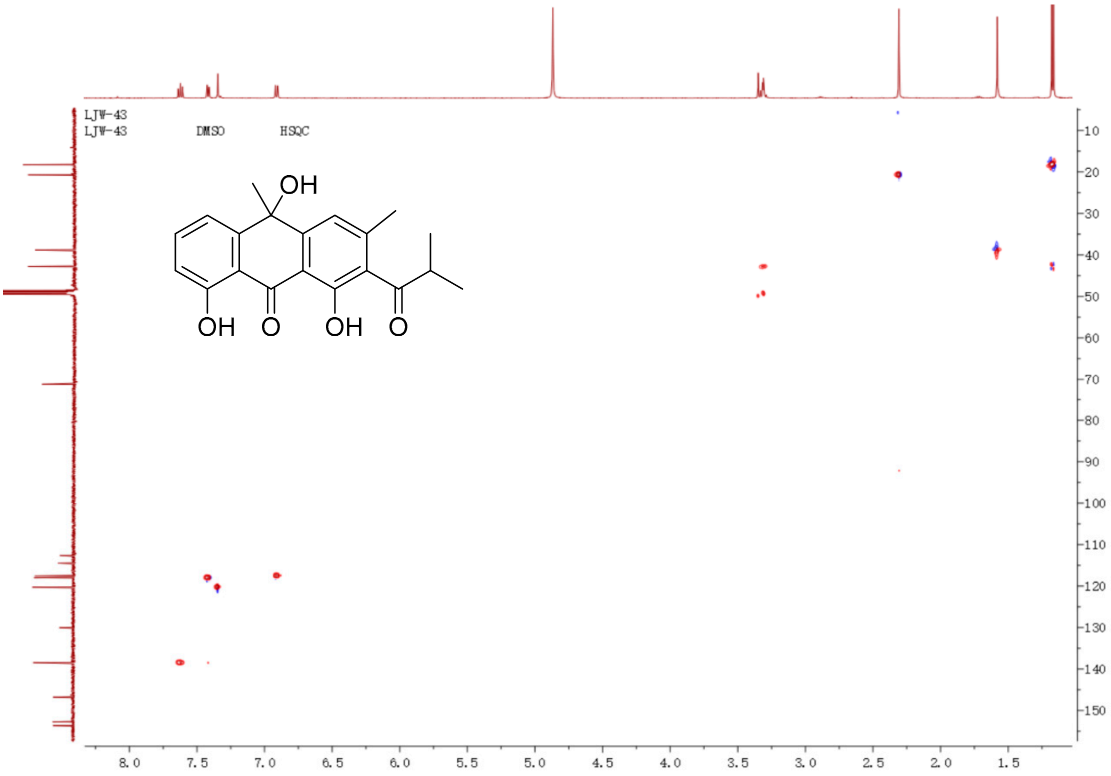

Figure S5. HMBC spectra of **3/4** in DMSO-*d6*.

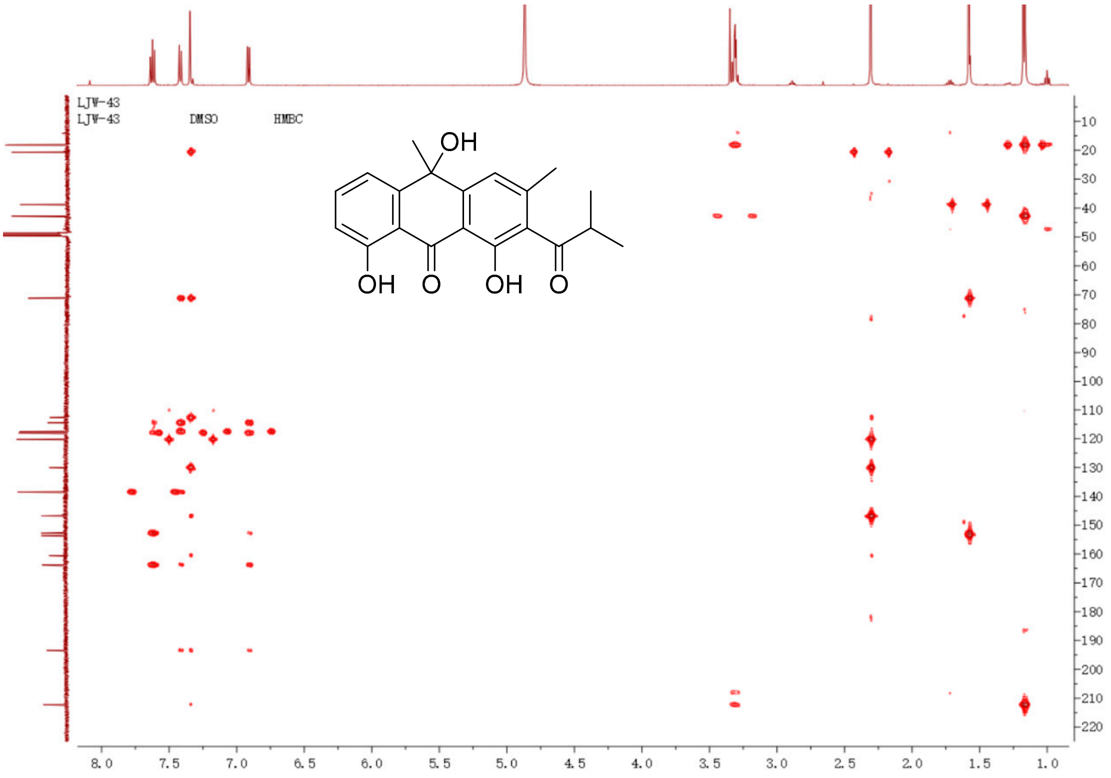

Figure S6. HRMS report of **3/4** in DMSO-d6.

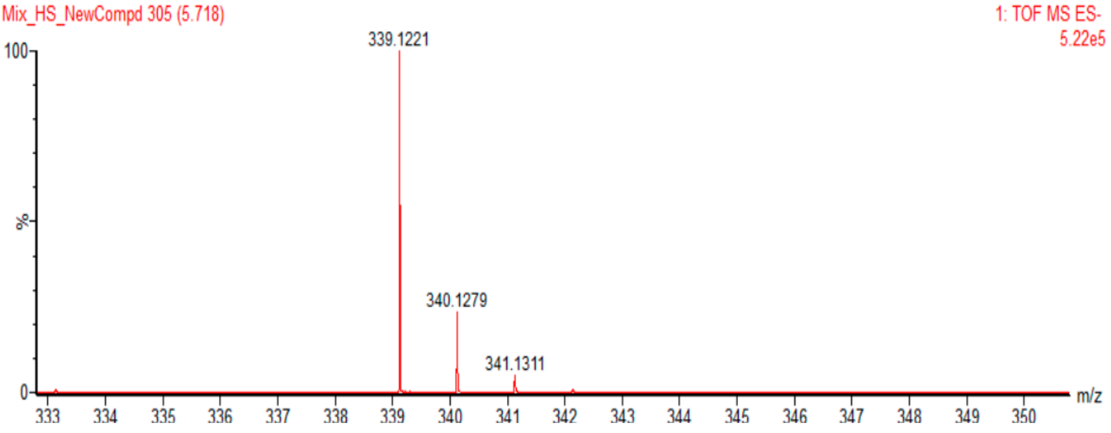

Figure S7. ^{1}H NMR spectrum of **5/6** in MeOD.

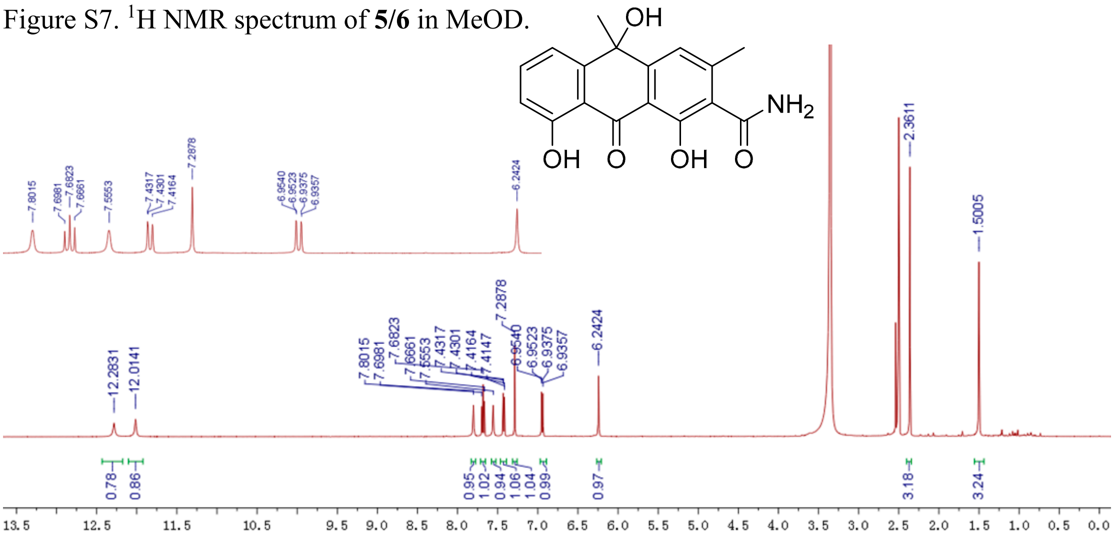

Figure S8. ^{13}C and DEPT NMR spectra of **5/6** in MeOD.

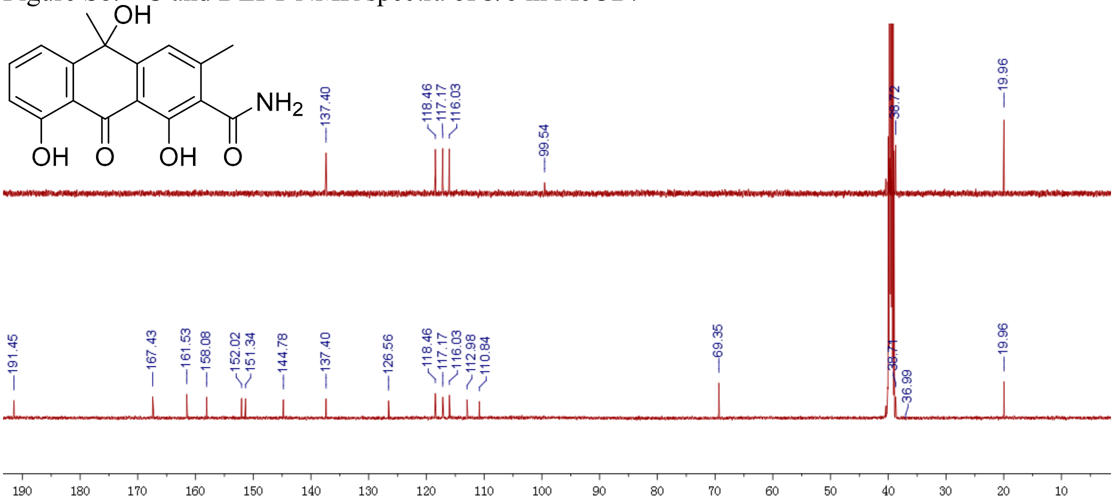

Figure S9. HMBC spectrum of **5/6** in MeOD.

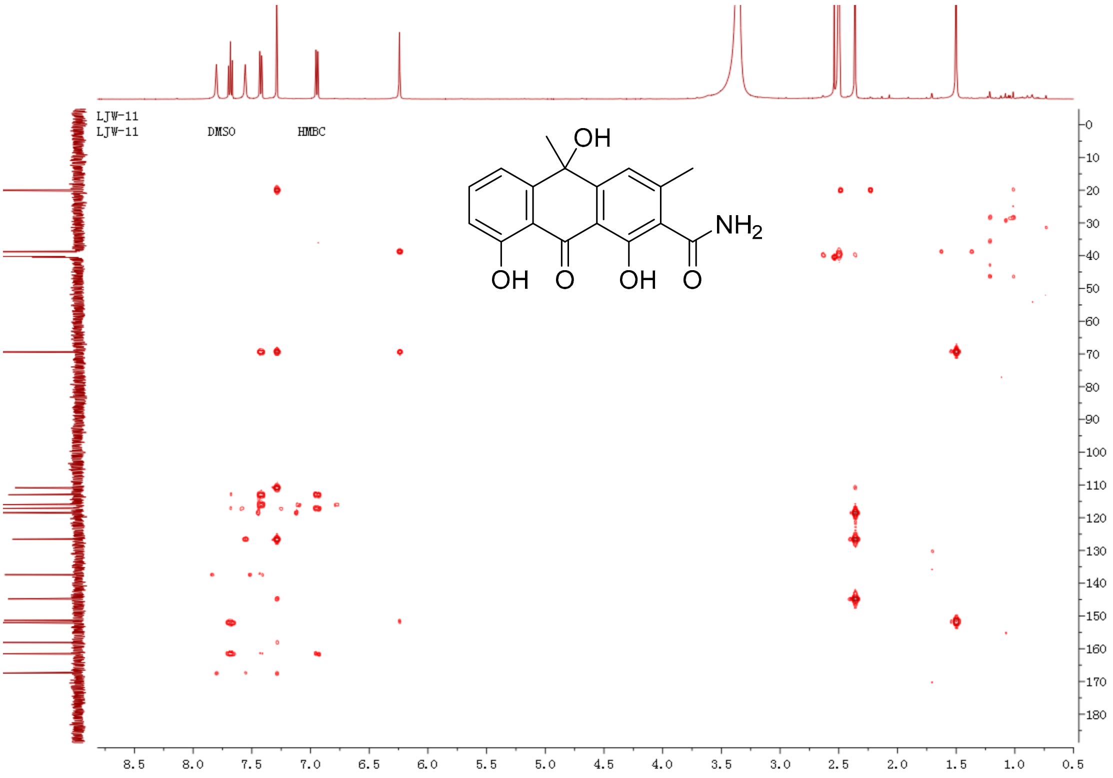

Figure S10. HRESIMS report of **5/6**.

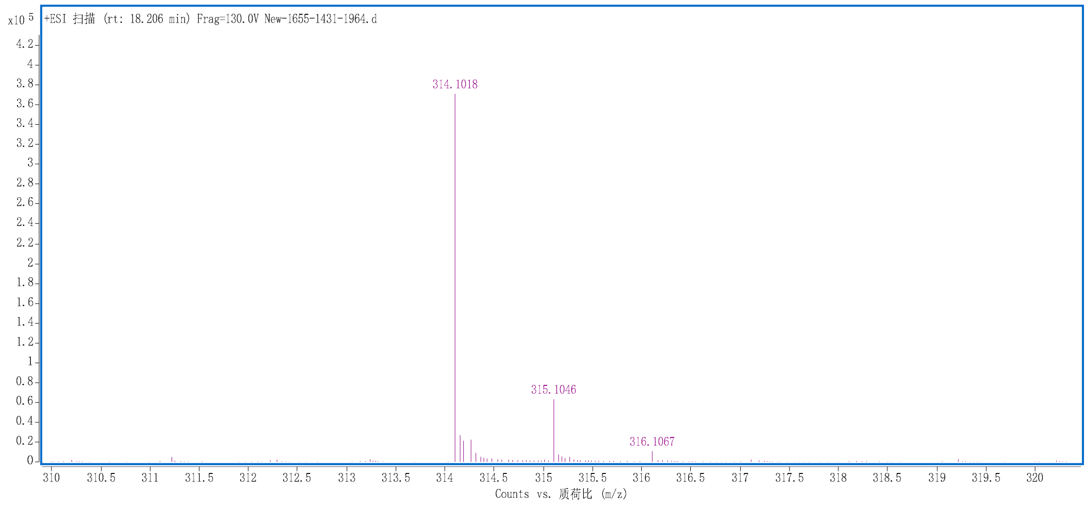

Figure S11. ^{1}H NMR spectrum of **7** in MeOD.

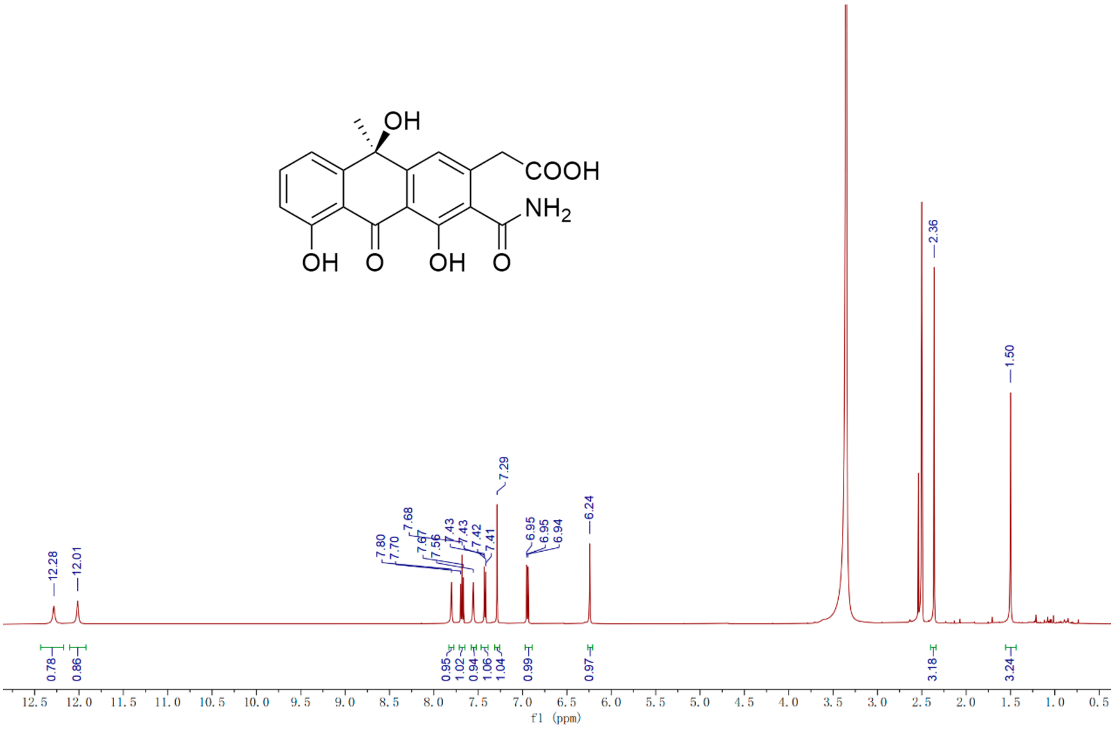

Figure S12. ^{13}C and DEPT NMR spectra of **7** in MeOD.

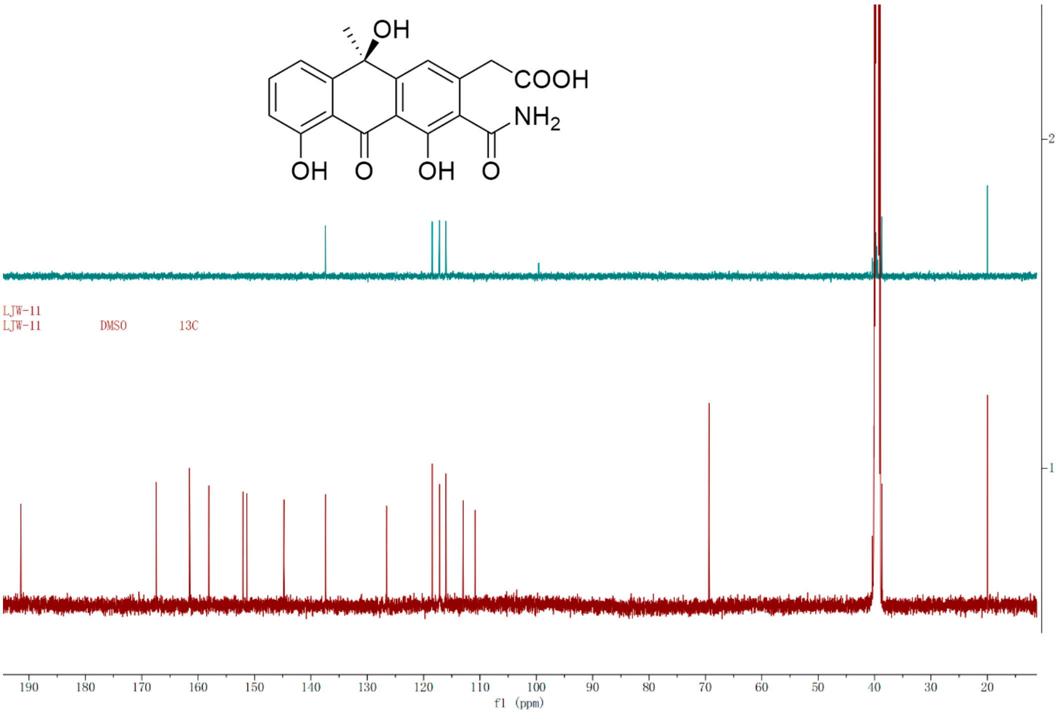

Figure S13. HMBC spectrum of **7** in MeOD.

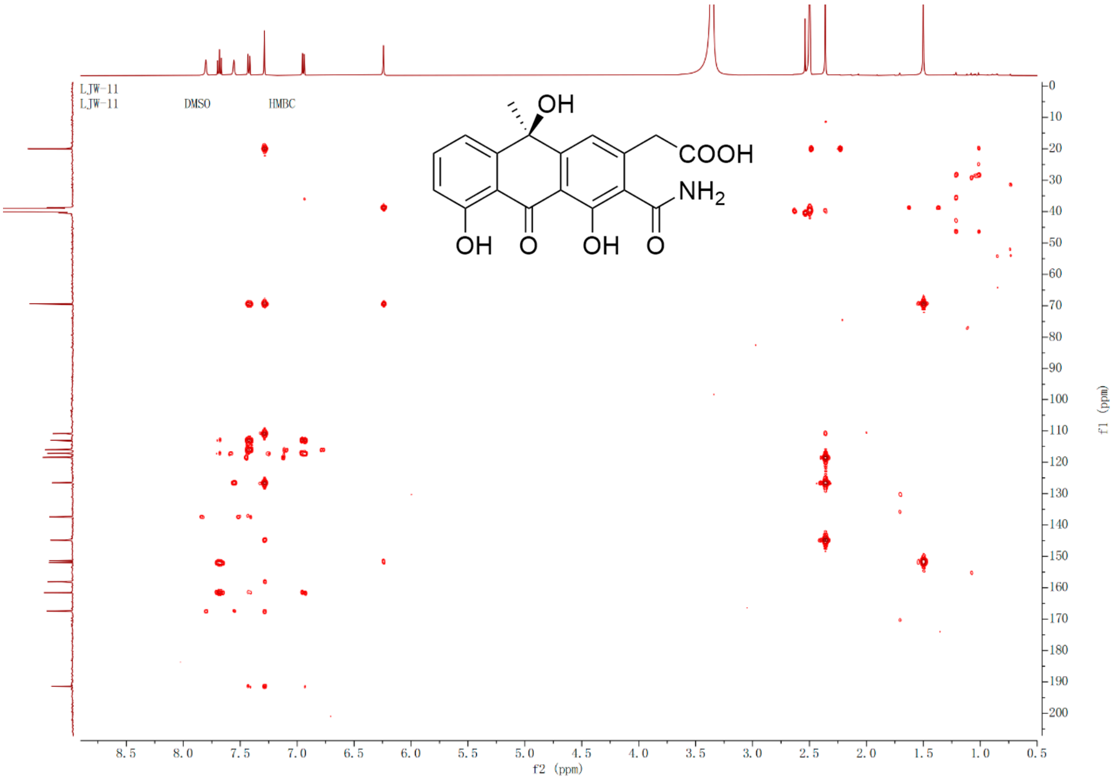

Figure S14. HRESIMS report of **7**.

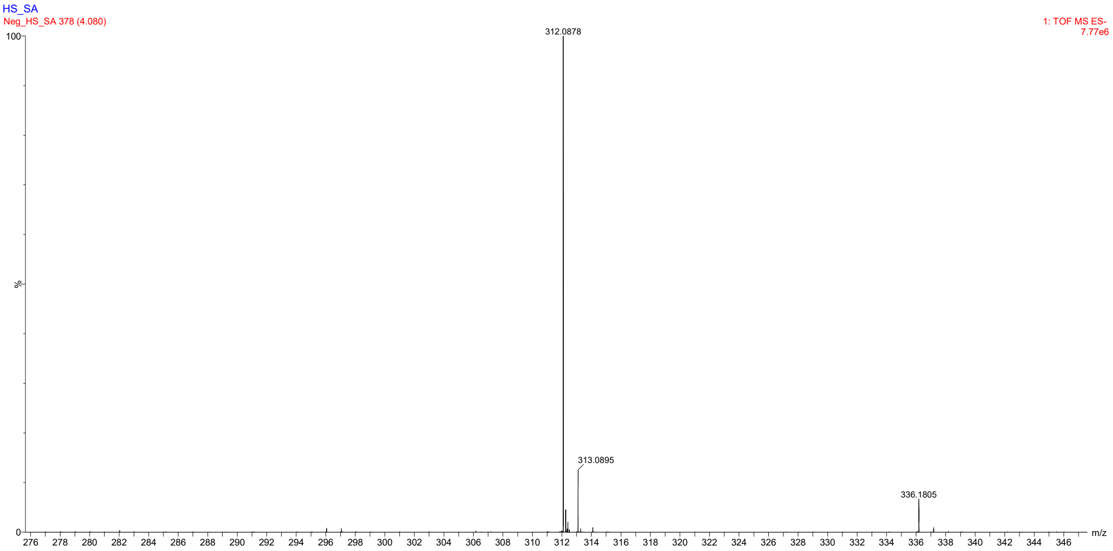

Figure S15. ^{1}H NMR spectrum of **8** in DMSO-d6.

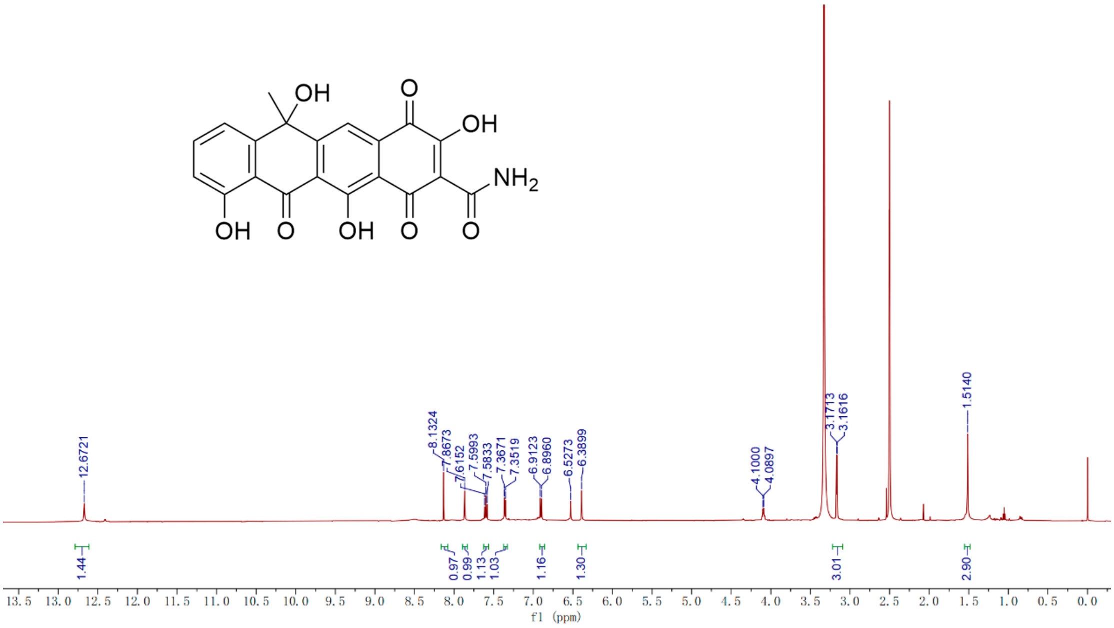

Figure S16. ^{13}C NMR spectrum of **8** in DMSO-d6.

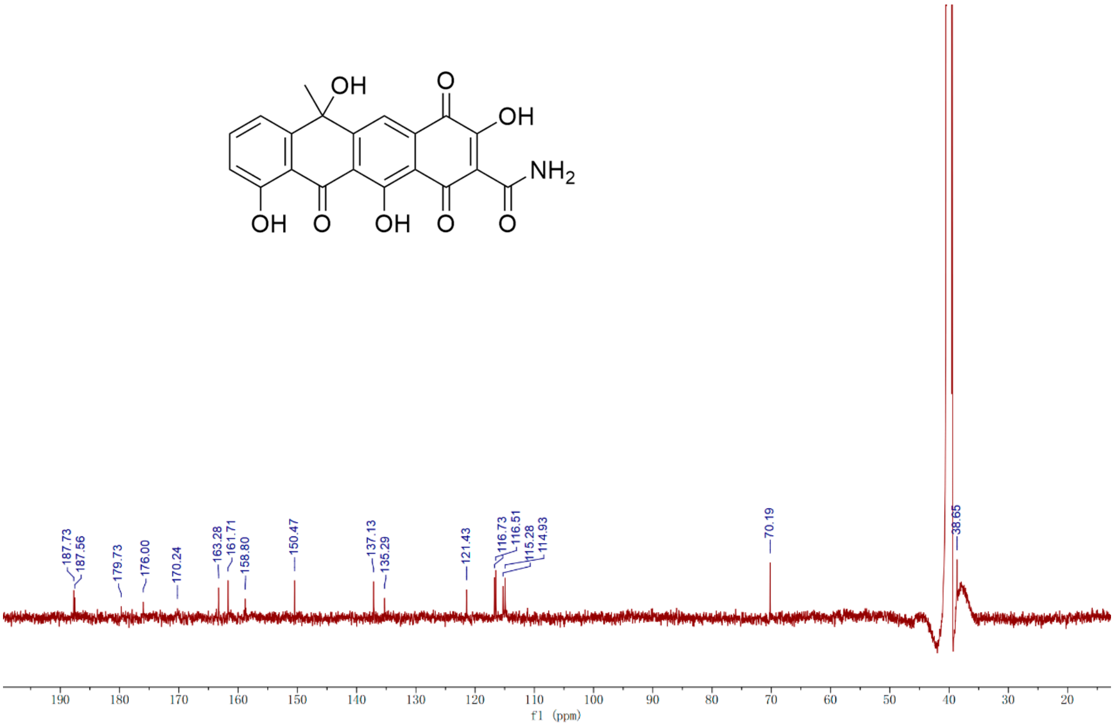

Figure S17. Comparison of experimental ECD spectra of compounds 1, 2 and 8.

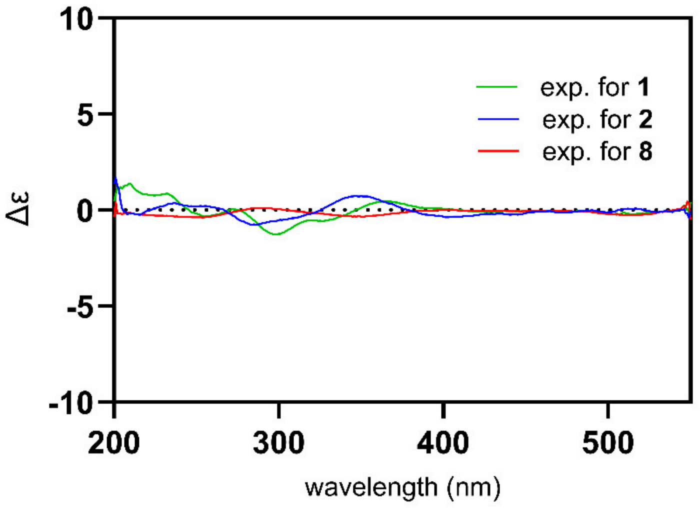

Figure S18. HRESIMS report of **8**.

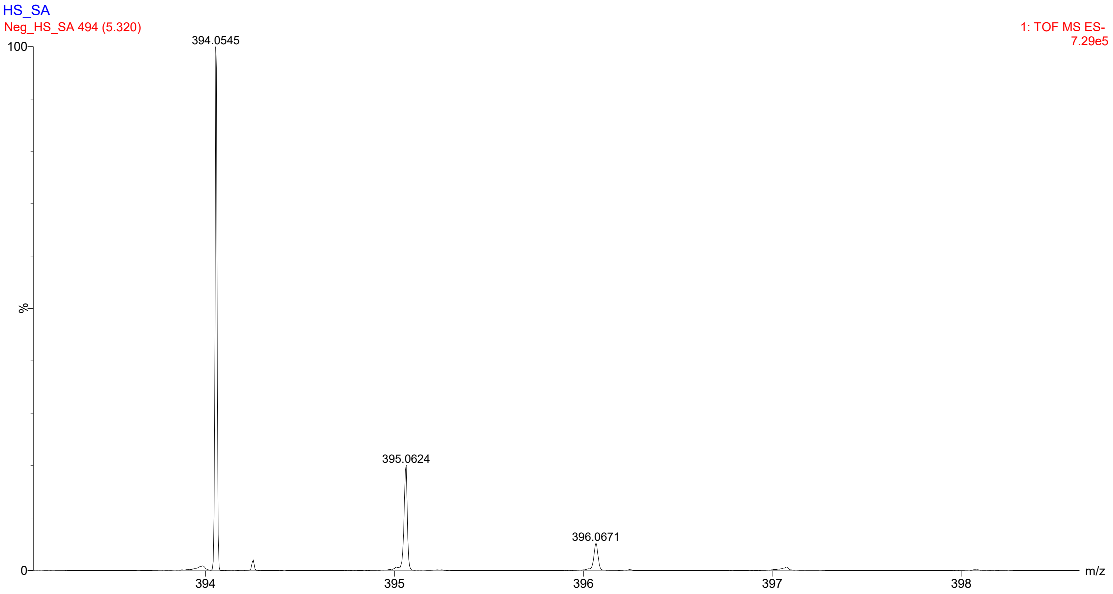

Supplement: Supplementary file 1 [file ijms-26-07801-s001.zip › ijms-3806671-supplementary.pdf]
